# Supplementary material for: Unsupervised learning on spontaneous retinal activity leads to efficient neural representation geometry
Source: ArXiv. 2023 Dec 5:arXiv:2312.02791v1. Preprint. [Version 1] (PMC10723543)
Supplement: Supplement 1 [file NIHPP2312.02791V1-supplement-1.pdf]

S0 Retina preparation and epifluorescent macroscope calcium imaging of retinal waves

We obtain real retinal wave movies using the following procedure. Mice aged postnatal day 8 to 11 (P8 to P11) are deeply anesthetized with isoflurane inhalation and euthanized by decapitation. Eyes are immediately enucleated and retinas are dissected at room temperature under infra-red illumination in oxygenated (95% O_2 / 5% CO_2) ACSF (in mM, 119 NaCl, 2.5 KCl, 1.3 $MgCl_2$, 1 K_2HPO_4, 26.2 $NaHCO_3$, 11 D-glucose, and 2.5 $CaCl_2$). Cuts along the chloride fissure are made prior to isolating the retina from the retinal pigmented epithelium. These cuts are made to precisely orient the retina. The isolated retina is mounted whole on filter paper with the photoreceptor layer side down, and transferred in a recording chamber of a microscope for subsequent imaging. The whole-mount retina is continuously perfused (3 mL/min) with oxygenated ACSF media at 32 to 34°C for the duration of the experiment. Epifluorescent whole-retina calcium imaging is obtained using transgenic mice that express GCaMP6s in all retinal ganglion cells (Vglut2::GCaMP6s). Waves are imaged on a custom built macroscope (4× 0.28 NA objective, a 498Hz kinetix camera, 4.7 mm × 4.7 mm FOV, and 1.5 µm/pixel) controlled by µManager 2.0 software. The oriented retina is mounted onto nitrocellulose filter paper (Millipore) for a darker background. Waves are imaged for 30 minutes at a 12.5 Hz frequency and pixels are binned 4 × 4 for a resolution of 5.9 µm/pixel. GCaMP6s excitation is evoked with a 476 nm LED.

S1 Analysis of retinal wave manifolds

A wave manifold as described in Fig. S1 is defined from a set of 50 frames from a randomly chosen single wave event in the original, unshuffled wave movie. We consider 50 such manifolds for all such metrics computed in Fig. S1 across the five pre-training conditions. Explained variance is the number of dimensions in feature space that account for 90% of the variance in the frames considered for manifold analysis. As expected, networks pre-trained on unshuffled waves yield the highest capacity amongst all pre-training conditions for the wave manifolds. Interestingly, the manifolds for real retinal waves appear to have higher capacity at all layers compared to those for simulated waves. This may be due to the smaller size of the real retinal waves dataset, which could lead to less variability across frames than in the simulated dataset. This explanation is consistent with the fact that the PR and EV for simulated waves is higher than for real waves. The trend in correlation, PR, and EV for both real and simulated wave manifolds reflects that observed in the task manifolds (Fig. 6), whereby the networks pre-trained on unshuffled waves maintain higher feature dimensionality and lower center correlation than the random networks, without producing a dimensionality explosion like the networks pre-trained on temporally shuffled waves.

S2 Simulation capacity

We report the correspondence between real and simulation capacity in the last activation layer (Fig. S2). We observe a high degree of correspondence between these values with exception of the overestimation of α_c for the color change manifold in networks pre-trained on simulated retinal waves (second row, third column). We also note that for retinal wave manifolds, calculation of α_{sim} is numerically unstable in the last activation layer for networks not trained on unshuffled waves (fourth column). This may occur when the manifold capacity is low relative to the feature dimension N, resulting in poor separability. For this reason, we instead report the values of α_c and α_{sim} for the wave manifolds in the projector layer (see Sections S4 for more details).

S3 Explained variance of task manifolds

We measure explained variance by the number of dimensions in feature space that account for 90% of the variance in the examples considered for manifold analysis (Fig. S3). The trend in EV in all tasks reflects that observed in center correlation and PR (Fig. 6), whereby the networks pre-trained on unshuffled waves maintain higher feature dimensionality and lower center correlation than the random networks, without producing a dimensionality explosion like the networks pre-trained on temporally shuffled waves.

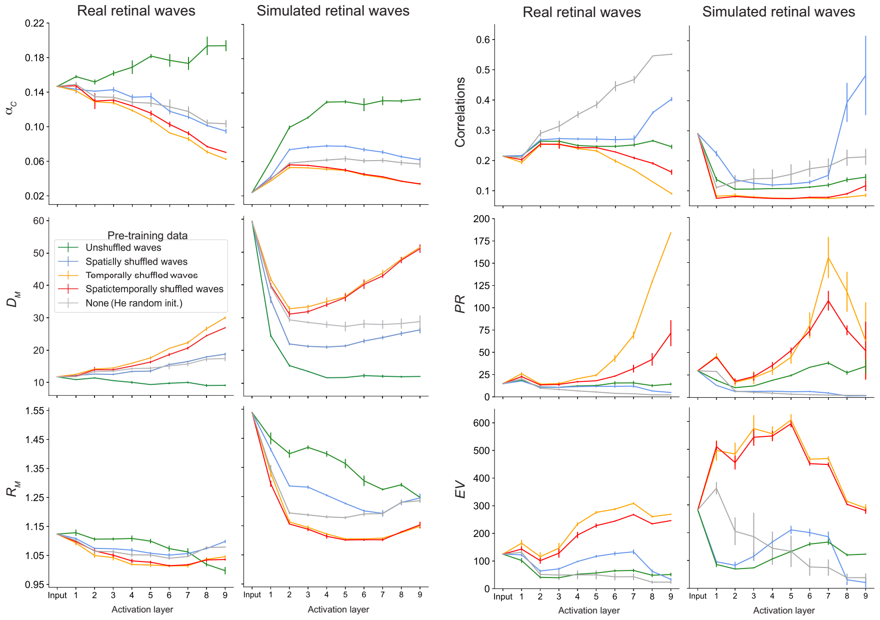

Figure S1: **Changes in (unshuffled) wave manifolds over network layers.**

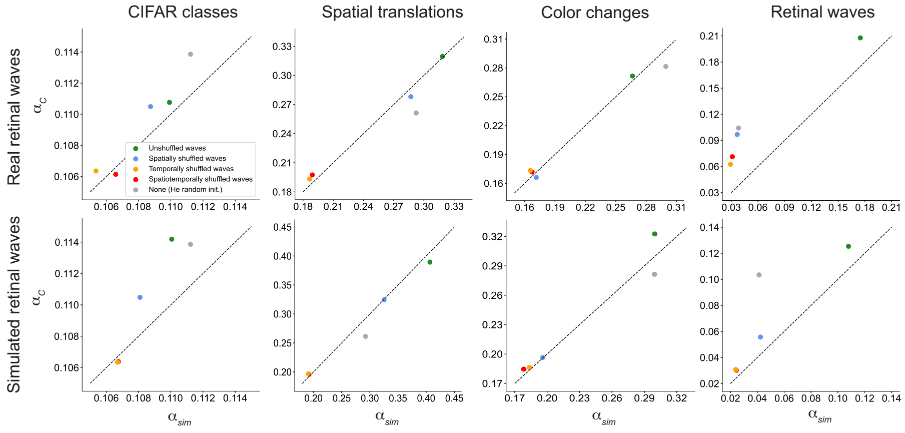

Figure S2: **Correspondence between theoretical and simulation capacity.** Each point represents mean over three random network initializations at the last activation layer, with the exception of the retinal wave manifolds (last column), where the measurements are taken in the projector layer. Dotted gray line denotes exact match between α_c and α_{sim}.

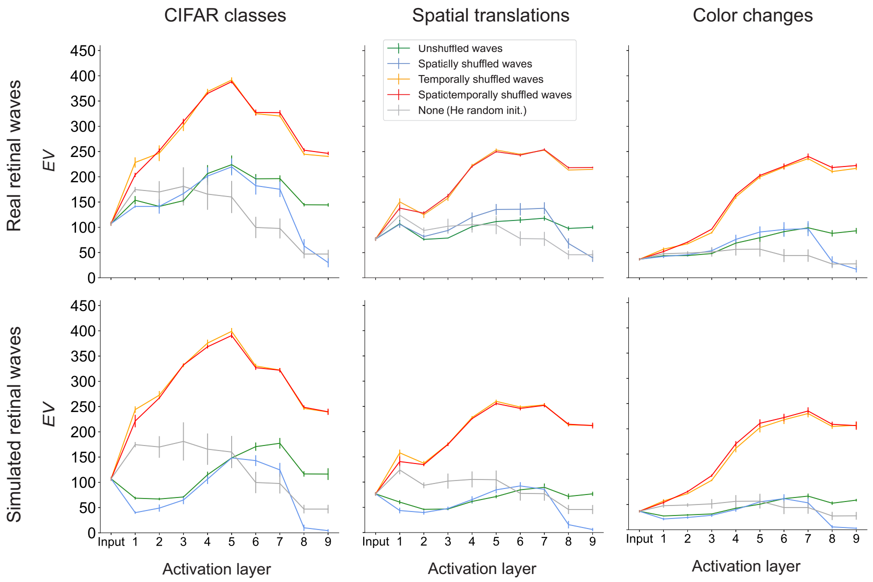

Figure S3: **Changes in explained variance over network layers.** y-axis denotes the number of feature dimensions that account for 90% of data variance.

S4 Model architecture and activation extraction

For all pre-training, we use a ResNet-18 network [58] (without the fully connected classification layer) followed by a projector layer. The projector consists of three linear layers with 8192 output units. The first two linear layers in the projector are each followed by a batch normalization layer and ReLU activations. The ResNet-18 backbone without the classification layer is sometimes referred to in self-supervised learning as an "encoder", and the outputs of the projector layer are referred to as "embeddings" [32]. The SimCLR loss is computed on the embeddings during pre-training, and during task training, the projector is swapped out with a 512×10 linear readout layer. This procedure of swapping out the projector has been shown empirically to be beneficial in transfer learning, where there is a misalignment between the pre-training and training tasks [59].

For all theoretical manifold quantities (α_c, D_M, R_M, correlation, PR, EV) the outputs of the intermediate ReLU activations in the encoder (for a total of 9 activation layers [58]) are extracted to analyze the internal representations of the task or wave manifolds at each layer. Due to the high computational cost, we only calculate the simulation capacity at the last ReLU in the encoder (unless otherwise stated; see Section S2 for details).

S5 Examples of real and simulated wave data

While we do not perform a direct quantitative comparison between the real and simulated retinal waves in this work, we present 3 representative examples from each dataset taken over a time period of about 18 sec. (Fig. S4). A key difference between the two datasets is that in the real retinal wave movies, the waves must terminate when they reach a boundary of the imaged retina (Fig. S5), but in the simulated retinal wave movies, the model "retina" is a uniform surface that extends beyond the field of view [31]. For this reason, in the simulated movies, the waves may continue past the frame. We partially adjust for this difference by setting the area parameter of the simulated retinal wave model as the average area of the calcium imaged retinas, though this adjustment does not account for any variations in wave characteristics induced by the retinal border. During pre-processing, both datasets are normalized to have a global mean and variance of 0 and 1, respectively. Both datasets are also given a new axis of size 3 and copied along this axis so that each color channel (RGB) of the network is pre-trained on the same data.

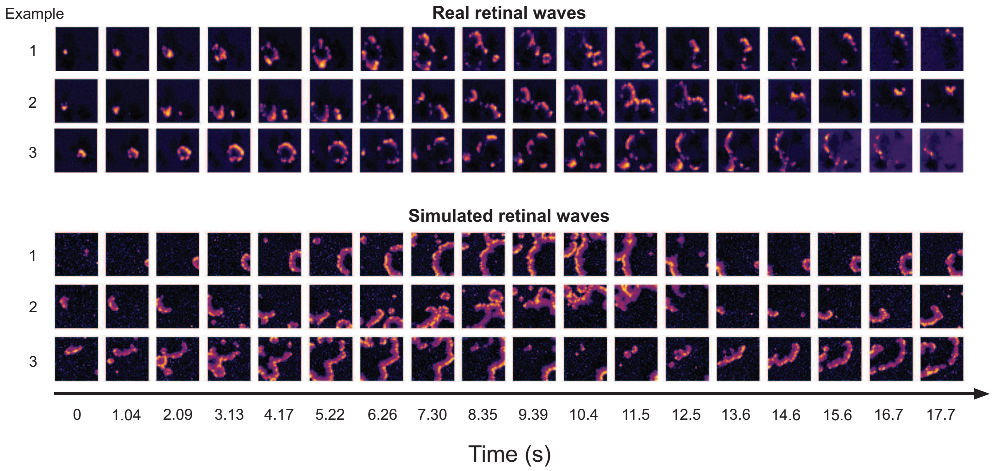

Figure S4: **Qualitative comparison of representative examples from real and simulated retinal wave movies.** For each example, every 12th frame is presented in order to visualize wave activity over longer a period of time.

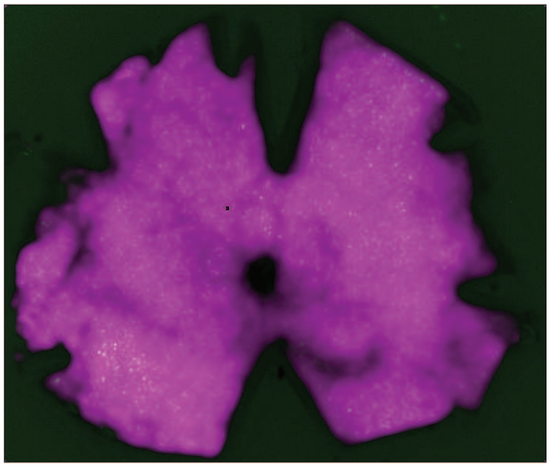

Figure S5: **Area of an isolated retina used to obtain real retinal wave data.** Retina shown in pink. Pixel-wise area calculated using open source Fiji software and then converting to metric units based on macroscope resolution.

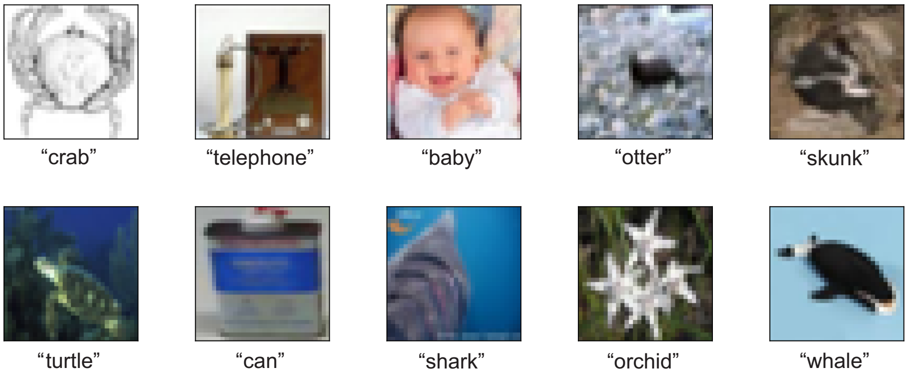

Figure S6: **Base images and labels for spatial translation and color change tasks.**

S6 Spatial translation and color change base images

For both spatial translation and color change task training and testing, we use the same base images shown in Fig. S6. These images are randomly sampled from CIFAR-100 [60]. Note that for generating the spatial translation and color change manifolds, we use a set of 50 randomly sampled base images that does not necessarily contain the base images used during task training and testing.
